# Sleep Quality and Sex-Specific Physical Activity Benefits Predict Mental Health in Romanian Medical Students: A Cross-Sectional Analysis

**DOI:** 10.3390/jcm14197121

**Published:** 2025-10-09

**Authors:** Catalin Plesea-Condratovici, Alina Plesea-Condratovici, Silvius Ioan Negoita, Valerian-Ionut Stoian, Lavinia-Alexandra Moroianu, Liliana Baroiu

**Affiliations:** 1 Morphological and Functional Sciences Department, Faculty of Medicine and Pharmacy, “Dunarea de Jos” University of Galati, 800008 Galati, Romania; catalin.plesea@ugal.ro; 2 Medical Department, Faculty of Medicine and Pharmacy, “Dunarea de Jos” University of Galati, 800008 Galati, Romania; valerian.stoian@ugal.ro; 3 Faculty of Medicine, University of Medicine and Pharmacy Carol Davila, 020021 Bucharest, Romania; 4 Pharmaceutical Sciences Department, Faculty of Medicine and Pharmacy, “Dunarea de Jos” University of Galati, 800201 Galati, Romania; lavinia.moroianu@yahoo.com; 5 Medical Clinical Department, Faculty of Medicine and Pharmacy, “Dunarea de Jos” University of Galati, 800008 Galati, Romania; liliana.baroiu@ugal.ro

**Keywords:** medical students, mental health, physical activity (PA), IPAQ, depressive symptoms, anxiety symptoms, walking, sleep quality, PSQI, sex differences, Romania

## Abstract

**Background**: Evidence on how everyday walking and sleep relate to mood in health profession students from Central–Eastern Europe remains limited. **Methods**: We conducted a cross-sectional study among 277 Romanian medical students. Data were collected using validated instruments for physical activity (IPAQ-SF), sleep quality (PSQI), and depressive/anxiety symptoms (HADS). Associations were examined using bivariate and multivariable regression models, including sex-stratified analyses. **Results**: In bivariate analysis, total physical activity was inversely correlated with depressive symptoms (ρ = −0.19, *p* < 0.001). However, in the multivariable model, this effect was not statistically significant after controlling for other factors. Poor sleep quality emerged as the dominant independent predictor of both depression (β = 0.37, *p* < 0.001) and anxiety (β = 0.40, *p* < 0.001). Walking time and frequency were specifically protective against depressive symptoms. Sex-stratified analyses revealed distinct patterns: female students benefited more from walking, whereas male students showed stronger associations between overall physical activity and lower depressive symptoms. **Conclusions**: Within the constraints of a cross-sectional design, this study provides novel evidence from Eastern Europe that sleep quality and physical activity are central to student mental health. Psychological benefits of walking appear sex-specific, and the null mediation finding suggests benefits operate via direct or unmodelled pathways. Sleep is a critical independent target for tailored, lifestyle-based strategies.

## 1. Introduction

Medical training is widely recognized as a demanding period associated with heightened psychological distress. Across diverse settings, medical students report substantial burdens of depressive symptoms, exceeding those observed in age-matched peers [1,2,3,4,5]. Multiple behavioral and psychosocial determinants have been implicated—including physical activity, sleep quality, and technology use—yet their relative contributions and interconnections remain incompletely understood, particularly in Central and Eastern Europe [1,2,6,7,8]. Clarifying which modifiable behaviors matter most, and for whom, is essential to inform targeted prevention within medical programs.

Physical activity (PA) is among the most consistent behavioral correlates of mental health. Observational syntheses and intervention trials indicate that higher habitual activity is associated with fewer depressive symptoms, and that structured exercise exerts small-to-moderate antidepressant effects [9,10,11]. The literature typically operationalizes PA at an aggregate level (e.g., total metabolic equivalent of task (MET)-min/week), which may obscure the contribution of light-intensity behaviors such as walking—an accessible, low-cost option that can be integrated into academic routines. Recent syntheses suggest that walking and higher daily step counts are associated with reduced depressive and anxiety symptoms [12,13], yet dedicated analyses in medical students remain scarce.

In parallel, sleep disturbance is highly prevalent among medical students and demonstrates robust associations with depression [14,15,16]. Conceptually, sleep may function as a mechanistic bridge linking activity patterns to mental health: regular activity improves sleep continuity and quality, whereas inadequate sleep impairs mood regulation and heightens negative affect [17,18,19]. A second candidate pathway frequently discussed in student populations is screen time/social media use, which may relate to sedentary behavior, circadian disruption, and internalizing symptoms; however, its explanatory value is modest and heterogeneous across studies [20,21].

A further determinant frequently examined is body mass index (BMI). While some population-based analyses describe a non-linear (U-shaped) association with depressive symptoms, the magnitude and consistency of BMI–mental health links in student cohorts are uncertain and may shrink once behavioral and psychosocial correlates are modeled [22,23]. Because medical students typically cluster within a narrow BMI range, studies may be underpowered to detect curvilinear effects; separating BMI from lifestyle covariates (sleep, activity) is therefore important. Additionally, potential sex differences warrant attention: women and men often engage in different activity profiles (e.g., walking vs. vigorous exercise) and may exhibit distinct psychological responses, implying value in sex-tailored recommendations [24,25].

Against this backdrop, there is a notable evidence gap in Romanian/Eastern European medical students integrating: (i) total physical activity and walking-specific metrics, (ii) sleep quality and screen time as candidate mediators, (iii) formal sex-stratified analyses, and (iv) explicit tests for BMI non-linearity alongside covariate adjustment. Addressing these gaps is timely for curriculum-level prevention strategies that are realistic to implement acknowledging the inherent limitation of causal inference within this cross-sectional framework.

To our knowledge, this is among the first studies from Central–Eastern Europe to jointly examine everyday walking, sleep quality, gaming time, and mood symptoms in health-professions students. By focusing on simple, scalable exposures (walking minutes; PSQI sleep quality), our findings offer context-specific evidence for a region that remains underrepresented in the literature.

## 2. Materials and Methods

### 2.1. Study Design and Participants

We conducted a cross-sectional study in Romanian medical students. All enrolled students who consented completed a standardized online survey that included anthropometrics, lifestyle, and validated psychometric instruments. Data collection for this study took place at the start of the fall semester of the 2024–2025 academic year. An online survey was administered to students using the PsyToolkit platform [26,27]. An a priori power analysis was conducted using G*Power 3.1.9.7 [28,29] to determine the required sample size to detect a medium effect size (f2 = 0.15) for our primary multivariable regression models, with a power of 0.80 and an alpha of 0.05. Given that our models included 9 predictors, this analysis indicated that a minimum sample size of 114 participants would be needed. The analytic sample comprised *N* = 277 students with complete outcome and exposure data for the primary analyses, which exceeded the a priori target and provided sufficient power to detect a small-to-medium effect.

The study adhered to the Declaration of Helsinki and national regulations. Ethical approval was obtained from Galaţi College of Physicians (No. 1165/29.11.2024) and electronic informed consent was obtained from all participants.

### 2.2. Measures

- Anthropometrics

Participants self-reported weight (kg) and height (cm); BMI was computed as kg/m^2^. Values ≤ 12 kg/m^2^ were considered implausible and set to missing a priori. BMI was modeled continuously (kg/m^2^) and with a quadratic term (BMI^2^) to test non-linearity. For descriptive context, World Health Organization (WHO) categories were reported (underweight, normal weight, overweight, obese).

- Physical activity (PA)

PA was assessed with the International Physical Activity Questionnaire—Short Form (IPAQ-SF) [30]. Following the standard scoring protocol, we calculated Total MET-minutes/week using 8.0 METs for vigorous, 4.0 METs for moderate, and 3.3 METs for walking, and derived IPAQ categories (Low/Moderate/High) using established thresholds. Because light-intensity activity was of specific interest, we also analyzed daily walking time (minutes/day) derived from the walking items of IPAQ.

- Mental-health outcomes

Symptoms of depression and anxiety were measured using the Hospital Anxiety and Depression Scale (HADS); subscales for depression and anxiety (HADS-D and HADS-A) range 0–21 (higher = worse) [31]. Outcomes were analyzed continuously.

- Sleep

Sleep quality was measured with the Pittsburgh Sleep Quality Index (PSQI); the global score (0–21; higher = worse) was used in analyses, with PSQI > 5 indicating poor sleep for descriptive reporting [32].

- Psycho-social covariates and screen time

Self-esteem was assessed with the Rosenberg Self-Esteem Scale (RSES) [33] (higher = better). Daily social-media time and daily gaming time were self-reported (hours/day). Age (years) and sex (female vs. male) were included as demographic covariates.

### 2.3. Data Preparation

Analyses followed a prespecified plan prior to modeling. Data were inspected for range and logic inconsistencies. Pairwise deletion was used for bivariate statistics and listwise deletion for multivariable models and mediation. Primary models used raw BMI; for descriptive purposes we flagged implausible BMI values (≤12 or >40 kg/m^2^) and excluded them only from Table 1 (BMI *n* = 274). Primary multivariable models retained *n* = 277; in sensitivity analyses excluding BMI < 16 or >40 kg/m^2^, conclusions were unchanged.

### 2.4. Statistical Analysis

All tests were two-sided with α = 0.05. Analyses were conducted in Python 3.11. Normality was examined by Shapiro–Wilk (primary) and D’Agostino’s K^2^ (*n* ≥ 20). No raw continuous variable met normality at α = 0.05; hence, bivariate analyses used non-parametric tests (Spearman’s ρ; Mann–Whitney U/Kruskal–Wallis with Dunn–Holm), and multivariable inference relied on OLS residual diagnostics with conventional standard errors; conclusions were unchanged in sensitivity analyses using HC3 robust standard errors.

Descriptive statistics: Means, standard deviations, and distributional summaries were computed for continuous variables; counts and percentages for categorical variables.

Bivariate associations. We estimated Spearman correlations (ρ) between BMI, Total MET-minutes/week, daily walking time, and HADS-D/HADS-A. For IPAQ categories (Low/Moderate/High), we compared HADS scores using Kruskal–Wallis tests.

Multivariable models: We fit ordinary least squares (OLS) regressions for HADS-D and HADS-A, including BMI, BMI^2^, Total MET-minutes/week, sex, age, PSQI, social-media time, gaming time, and RSES as covariates. We report unstandardized coefficients, standard errors, *p*-values, 95% CIs, and model fit (R^2^). For visualization (forest plots), predictors were standardized (z-scores) and standardized betas were displayed with 95% CIs.

Walking-specific analyses: We examined walking indicators (e.g., minutes/day, walking METs, total walking minutes) against HADS-D/HADS-A via Spearman ρ and summarized results in a dedicated table.

Sex-stratified analyses: To explore potential effect heterogeneity, we computed sex-stratified correlations (female vs. male) for BMI, Total MET-minutes/week, and daily walking time with HADS-D/HADS-A and presented them as a table and forest-style figure.

Mediation analysis: We tested a parallel mediation model with walking time as predictor (X), PSQI global score and social-media time as mediators (M1, M2), and HADS-D or HADS-A as outcomes (Y), adjusting for age, sex, and BMI. All variables (except sex) were standardized prior to modeling. The a-paths (X→M_1_/M_2_) and b/c′ paths (M_1_/M_2_ and X→Y) were estimated via OLS. Indirect effects (X→M_1_→Y; X→M_2_→Y) and total indirect were quantified using non-parametric bootstrap (≥1200 resamples) with percentile 95% CIs. An indirect effect was considered significant when the 95% CI excluded 0. We summarize a, b, c, c′ coefficients and indirect effects in the mediation table and depict the model schematically.

Multiple comparisons and robustness: We emphasize effect sizes and compatibility intervals rather than multiplicity-adjusted *p*-values, consistent with our a priori focus on a limited set of theoretically motivated relationships (PA/Walking ↔ HADS; mediation via PSQI vs. social media). Robustness was examined via sensitivity exclusions for BMI extremes and by comparing crude vs. adjusted estimates.

## 3. Results

### 3.1. Sample Characteristics

**Figure 1:**
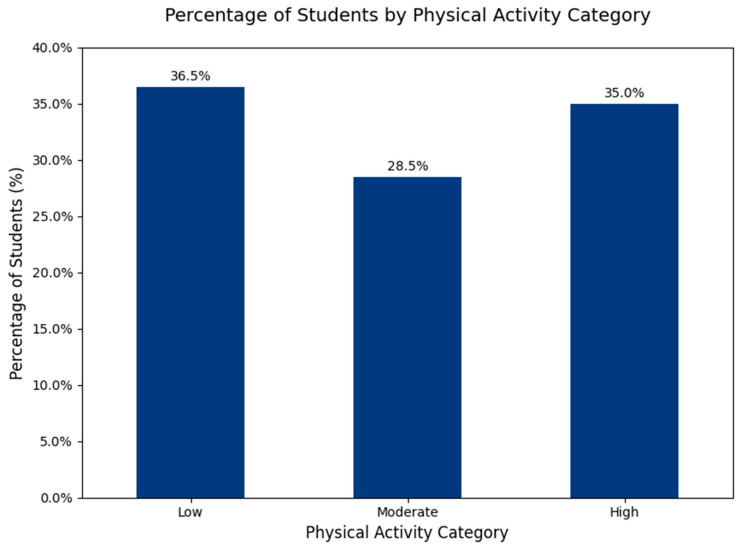
Students Distribution by IPAQ Categories.

A total of 277 medical students were analysed. The mean age was 23.61 years (standard deviation (SD) = 8.23). Regarding ponderal status, 11.19% were underweight, 66.79% normal weight, 17.33% overweight, and 4.69% obese. According to the IPAQ classification, 36.50% of participants were categorized as having low physical activity, 28.50% moderate, and 35.00% high (Figure 1). Two-thirds of the sample reported poor sleep quality (PSQI > 5, 67.15%). Descriptive statistics for the main continuous variables are presented in Table 1.

### 3.2. Sleep and Physical Activity Descriptives

**Figure 2:**
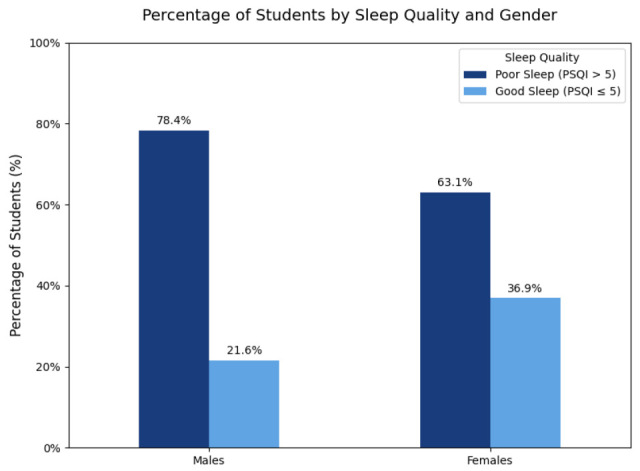
Prevalence of Poor Sleep Quality Among Students by Sex.

Mean PSQI was 7.20 ± 3.43 (median 7 [5–9]); 67.15% (186/277) met the threshold for poor sleep (PSQI > 5). By sex, poor sleep prevalence was 78.38% in males vs. 63.05% in females (Figure 2) (χ^2^ *p* = 0.0239), whereas mean PSQI did not differ statistically (7.57 ± 2.83 vs. 7.06 ± 3.62, *p* = 0.114).

Total physical activity (IPAQ-SF) showed marked skew: median 2079 MET-min/week; interquartile range (IQR) 132–4551 mean 3309 ± 4260. Overall, 68.6% (190/277) met WHO’s minimum (≥600 MET-min/week), with no sex difference on prevalence (60.80% males vs. 71.43% females; *p* = 0.124). Daily walking time averaged 80.2 ± 68.3 min/day (median 80 [0–135]), with no statistically significant difference between females (83.7 ± 65.9) and males (70.4 ± 74.1; *p* = 0.1151). IPAQ categories were Low 36.5% (*n* = 101), Moderate 28.5% (*n* = 79), and High 35.0% (*n* = 97).

Table 2, Table 3 and Table 4 summarize PSQI and IPAQ distributions overall and by sex (with Mann–Whitney U/χ^2^ tests), and the prevalence meeting WHO’s minimum (≥600 MET-min/week).

### 3.3. Bivariate Associations

Spearman correlations were conducted to examine associations between BMI, physical activity, and depressive/anxiety symptoms (Table 5).

- Physical activity and depression: Total MET-min/week correlated negatively and significantly with HADS-D (ρ = −0.19, *p* = 0.0015), indicating that more active students reported fewer depressive symptoms.
- Physical activity and anxiety: The association with HADS-A was non-significant (ρ = 0.00, *p* = 0.9835).
- BMI and symptoms: BMI showed no significant correlation with HADS-D (ρ = 0.09, *p* = 0.1486) or HADS-A (ρ = −0.07, *p* = 0.2560).

### 3.4. Group Comparisons by IPAQ Categories

We compared mean symptom scores across IPAQ levels (Low, Moderate, High) using Kruskal–Wallis tests (Table 6).

- HADS-D: significant differences were found (*p* = 0.024). Students with low physical activity reported higher depressive symptoms compared to those with moderate or high activity.
- HADS-A: no significant group differences were observed (*p* = 0.92).

### 3.5. Multivariable Regression Models

We estimated OLS regression models for HADS-D and HADS-A including BMI, BMI^2^, Total MET, sex, age, PSQI TOTAL, time spent on social media, time gaming, and RSES (Table 7 and Table 8).

- Depressive Symptoms (HADS-D): The full model explained 23.61% of the variance in HADS-D scores (R^2^ = 0.236, F(9, 267) = 9.168, *p* < 0.001). Significant predictors included poor sleep quality (PSQI_TOTAL; β = 0.368, *p* < 0.001) and being female (β = −1.621, *p* = 0.001), which had a negative effect on depressive symptoms. Time spent on social media was also a significant negative predictor (β = −0.377, *p* = 0.014). Physical activity (Total MET) retained a borderline inverse effect (β = −0.0001, *p* = 0.072). Neither BMI nor BMI^2^ showed a significant effect.
- Anxiety Symptoms (HADS-A): The full model explained 21.2% of the variance in HADS-A scores (R^2^ = 0.212, F(9, 267) = 7.979, *p* < 0.001). Significant predictors were poor sleep quality (PSQI_TOTAL; β = 0.397, *p* < 0.001), which was positively associated with anxiety, and self-esteem (RSES; β = −0.126, *p* = 0.001), which was inversely associated. Neither total physical activity nor BMI showed a significant independent effect.

### 3.6. Walking Time Analyses

To further disentangle the role of light physical activity, we examined specific IPAQ walking-related variables in relation to depressive and anxiety symptoms (Table 9).

- Associations with Depressive Symptoms (HADS-D): All three walking variables showed a significant negative correlation with depressive symptoms. The strongest association was with the number of days walked per week (ρ = −0.19, *p* = 0.002), indicating that a higher frequency of walking was related to fewer depressive symptoms. Similarly, daily walking time (ρ = −0.15, *p* = 0.012) and total walking METs (ρ = −0.15, *p* = 0.010) were also negatively correlated with HADS-D scores.
- Associations with Anxiety Symptoms (HADS-A): In contrast, walking variables showed no statistically significant association with anxiety symptoms. The correlations were weak for days walked per week (ρ = −0.11, *p* = 0.067), daily walking time (ρ = −0.03, *p* = 0.634), and total walking METs (ρ = −0.04, *p* = 0.526).

The findings suggest that walking, in terms of its frequency, duration, and energy expenditure, serves as a protective factor against depressive symptoms in this sample, but does not have a similar protective effect against anxiety.

### 3.7. Gender-Stratified Analyses

**Figure 3:**
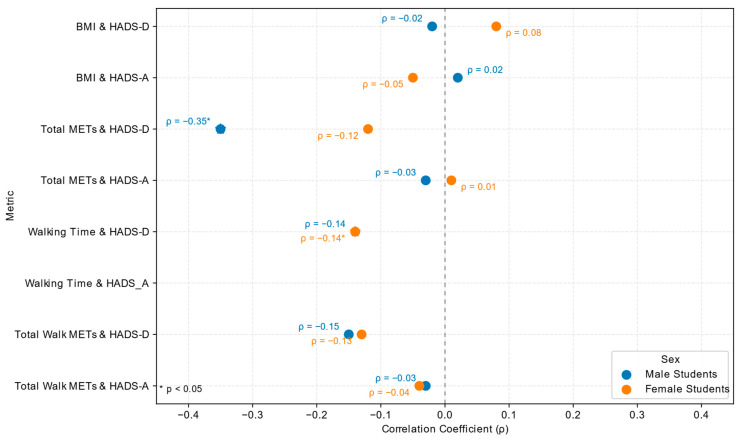
Sex-stratified Spearman correlations (ρ) between physical activity, BMI, and mental-health outcomes (HADS-D and HADS-A). Blue = male students; orange = female students. Star-shaped symbols (★) denote statistically significant correlations (*p* < 0.05). Negative ρ values indicate inverse relationships (higher activity or walking time associated with lower depressive symptom scores).

To explore potential sex differences, correlations between BMI, total physical activity, and walking time with depressive and anxiety symptoms were analyzed separately for males and females (Table 10, Figure 3).

The correlations between physical activity, BMI, and mental health symptoms show sex-specific patterns.

- Female Students: Among female students, walking time showed a significant negative correlation with depressive symptoms (ρ = −0.14, *p* = 0.0443), but no significant association with anxiety symptoms (ρ = −0.02, *p* = 0.7294). In contrast, total METs were not significantly associated with depression (ρ = −0.12, *p* = 0.0759) or anxiety (ρ = 0.01, *p* = 0.8675).
- Male Students: Among male students, the strongest association was observed for total METs and depressive symptoms, with a significant negative correlation (ρ = −0.35, *p* = 0.0021). Walking time did not significantly correlate with either depression (ρ = −0.14, *p* = 0.2239) or anxiety (ρ = −0.05, *p* = 0.6796).

These findings suggest a sex-specific pattern: female students appear to benefit more psychologically from light-intensity activity such as walking, while male students may derive greater psychological benefit from vigorous or overall higher-intensity physical activity.

### 3.8. Mediation Analyses

We estimated OLS regression models for HADS-D and HADS-A to explore parallel mediation pathways through sleep quality and social media time, adjusted for age, sex, and BMI.

For depressive symptoms, there was no significant total effect of walking time on HADS-D (β = −0.0052, *p* = 0.1082). Consistent with this, neither the direct path (β = −0.0029, *p* = 0.3405) nor the indirect paths via sleep quality (95% CI [−0.0037, 0.0015]) or social media use (95% CI [−0.0028, 0.0000]) were found to be significant. The results did not provide evidence for mediation as the effect of walking on depressive symptoms was non-significant throughout the model.

Similarly, for anxiety symptoms, there was no significant total effect of walking time on HADS-A (β = −0.0005, *p* = 0.9046). Both the direct path and the indirect paths through sleep quality and social media time were also non-significant.

### 3.9. Concise Summary of Results

Among 277 medical students, poor sleep was common (67.1%). In bivariate analyses, higher total physical activity related to fewer depressive symptoms (ρ = −0.19, *p* = 0.0015), whereas walking indicators were inversely associated with depression but not anxiety; BMI showed no associations. In multivariable models (R^2^ = 0.236 for HADS-D; R^2^ = 0.212 for HADS-A), sleep quality was the strongest independent correlate of both depression (β = 0.37, *p* < 0.001) and anxiety (β = 0.40, *p* < 0.001); total physical activity showed only a borderline inverse association with depression and was null for anxiety; BMI had no independent effect. Sex-stratified correlations suggested that females benefit more from walking, whereas in males higher total activity relates to fewer depressive symptoms. Parallel-mediation analyses via sleep and social-media time were null.

## 4. Discussion

### 4.1. Findings

This cross-sectional study investigated the relationship between physical activity, BMI, and depressive and anxiety symptoms in Romanian medical students. This research also examined the potential roles of sleep quality and screen time as contributing factors. The study aimed to clarify which modifiable behaviors are most influential for this specific population to inform targeted prevention strategies.

The finding of a negative correlation between overall physical activity and depressive symptoms among students is strongly supported by current evidence. Multiple large-scale studies and meta-analyses demonstrate that higher levels of physical activity are associated with lower depressive symptom scores in university and high school populations, with the relationship being dose-dependent—students with low physical activity consistently report higher depressive symptoms compared to those with moderate or high activity levels [9,35,36,37,38,39,40]. While our bivariate findings align with literature suggesting PA is a protective factor, this effect was not supported in our more comprehensive multivariable model. This suggests that the relationship between total PA volume and depression may be mediated or confounded by other variables, such as sleep quality, in this specific cohort. The literature also indicates that physical activity exerts its protective effect on depression through mediating factors such as self-esteem, positive psychological capital, and social support, which further reduce depressive symptoms [35,37]. Specific depressive symptoms most affected by physical activity include anhedonia and fatigue, with a monotonic dose–response relationship observed for these domains [41].

Regarding anxiety symptoms, the evidence is less consistent. While some studies report that physically inactive students have higher anxiety scores, the association between physical activity and anxiety is generally weaker and often not statistically significant after adjustment for confounders [38,42].

There is robust evidence that walking, even at light intensity and modest frequency, is associated with a significant reduction in depressive symptoms. Large-scale meta-analyses and cohort studies consistently demonstrate an inverse, curvilinear dose–response relationship: the greatest mental health benefits are observed when individuals move from no activity to some activity, with diminishing returns at higher levels of physical activity [9,12,43]. For example, accumulating a volume of physical activity equivalent to 2.5 h of brisk walking per week is associated with a 25% lower risk of depression, and even half that dose confers an 18% lower risk compared to inactivity [9].

Daily step count data further support these findings: adults who achieve 5000 or more steps per day report fewer depressive symptoms, and those exceeding 7500 steps per day have a 42% lower prevalence of depression compared to those with fewer than 5000 steps [12]. Importantly, these protective effects are evident with walking and do not require vigorous exercise, making walking a highly accessible and low-cost intervention.

Among college students, higher frequency and duration of walking are specifically correlated with lower depressive symptom scores and engaging in physical activity more than three times per week for 30–59 min is associated with a significantly lower detection rate of cognitive symptoms and suicidal ideation [36,39,44]. The relationship is partially mediated by improvements in sleep quality and behavioral activation, but the direct effect of physical activity remains substantial [36,44].

In summary, even light-intensity physical activity such as walking is a protective factor against depression, and increasing walking days per week and minutes per day can be recommended as a practical, evidence-based strategy for reducing depressive symptoms in students and adults.

Current evidence supports sex-specific recommendations for physical activity to support mental health in students. Multiple large-scale studies demonstrate that for female students, walking time and lower-intensity activities are more consistently associated with fewer depressive symptoms, while for male students, higher-intensity physical activity (such as moderate-to-vigorous exercise or activities with higher total metabolic equivalents) shows a stronger inverse relationship with depressive symptoms [45,46,47,48,49,50].

For females, walking more than once per week is associated with lower depression levels, and reallocating sedentary time to walking or moderate-intensity activity is beneficial [45,46]. In contrast, for males, engaging in strength or vigorous exercise more than once per week, or increasing overall moderate-to-vigorous physical activity, is linked to reduced depressive symptoms [45,46,48,49]. These patterns are consistent across diverse populations and are robust to adjustment for confounders.

The literature also highlights that the context and type of physical activity matter: leisure-time physical activity is more protective for mood than occupational or household activity, and the benefits of physical activity for mental health are greater when tailored to sex-specific preferences and physiological responses [50].

In summary, recommendations for physical activity to support mental health should be tailored by sex: emphasizing walking and moderate activity for females, and higher-intensity or strength-based activity for males, to optimize reductions in depressive symptoms among student populations.

Poor sleep quality is a strong, independent predictor of both depressive and anxiety symptoms among medical students, as consistently demonstrated in recent cross-sectional and longitudinal studies. Multiple analyses using validated instruments (e.g., Pittsburgh Sleep Quality Index, Depression Anxiety Stress Scales, Patient Health Questionnaire (PHQ-9), Generalized Anxiety Disorder 7-item scale (GAD-7)) show that poor sleep quality is directly associated with increased risk and severity of depression and anxiety, even after controlling for confounders such as sex, academic year, and psychosocial stressors [51,52,53,54,55,56,57,58,59,60,61].

Longitudinal data further support a bidirectional relationship: poor sleep quality predicts future onset and worsening of depressive and anxiety symptoms, and conversely, baseline depression and anxiety can worsen sleep quality over time [54,62]. Mediation analyses indicate that sleep quality often serves as a key mechanism linking stress, negative life events, and psychological distress to mental health outcomes in this population [52,53,57,59,60,61].

The clinical implication is that interventions targeting sleep quality—such as cognitive behavioral therapy for insomnia, sleep hygiene education, and resilience-building—may be effective strategies to mitigate depressive and anxiety symptoms in medical students. These findings reinforce the robust connection between sleep and mental health, underscoring the importance of routine screening and integrated management of sleep disturbances in this high-risk group.

Current evidence indicates that the association between body mass index (BMI) and depressive or anxiety symptoms is complex and context-dependent. Large population-based studies consistently report a U-shaped relationship, with both underweight and obesity associated with increased risk of depression, while normal BMI is associated with lower risk [63,64,65,66]. However, when confounding factors such as lifestyle, academic stress, and internet use are accounted for—particularly in narrow cohorts like medical students—the direct association between BMI and depressive or anxiety symptoms may attenuate or become non-significant [22,67]. It is important to acknowledge that the narrow distribution of BMI in our specific student population (median 22.41 kg/m^2^) may have reduced the power to detect subtle or curvilinear associations, which is a common limitation in similar homogenous samples.

In medical students, academic burnout and internet addiction have been shown to mediate the relationship between overweight/obesity and mental health symptoms, suggesting that lifestyle and psychosocial factors may be more influential than BMI alone in this population [22]. This finding challenges the generalizability of population-based associations to specific subgroups, where unique stressors and coping mechanisms may play a larger role.

Regarding screen time, while it is a significant predictor of depressive symptoms in some studies, its explanatory value is modest and inconsistent across different populations and study designs [65]. Screen time may interact with other factors such as social comparison, body image concerns, and academic stress, but it does not fully account for the variance in depressive symptoms.

In summary, the link between BMI and mental health is less clear in narrowly defined cohorts like medical students when lifestyle factors are considered, and screen time, although relevant, is a modest and inconsistent predictor of depressive symptoms.

While physical activity, including walking, is robustly associated with reduced depressive symptoms, mediation analyses have shown mixed results regarding the roles of sleep and social media use.

For sleep, some studies have found partial mediation, indicating that improvements in sleep quality can explain part of the mental health benefits of physical activity, but not all. For example, Kaseva et al. found that sleep problems mediated approximately 30–36% of the association between physical activity and depressive symptoms, but this effect was attenuated after adjusting for baseline depression, suggesting that other pathways are also important [68]. Similarly, Barham et al. reported that sleep health mediated only 19% of the association between physical activity and depression symptoms, indicating that the majority of the effect is direct or mediated by other factors [69].

Regarding social media use, Wang et al. demonstrated that physical activity is associated with lower depression and anxiety, and that high social media use is associated with worse mental health outcomes but did not establish social media use as a mediator between physical activity and mental health [70]. Systematic reviews further support that while social media use and sleep quality are independently associated with mental health, their roles as mediators in the physical activity–mental health relationship are limited or inconsistent [71,72].

Therefore, the finding that the beneficial effect of walking on mental health is not accounted for by improvements in sleep or alterations in social media use is supported by current evidence, which suggests that walking likely exerts its mental health benefits through direct physiological and psychological mechanisms, rather than primarily through changes in sleep or social media behaviors.

### 4.2. Theoretical and Practical Implications

The association between physical activity and mental health is indeed not a one-size-fits-all phenomenon. Recent evidence demonstrates that both the type and intensity of physical activity, as well as sex, can moderate mental health benefits. For example, large-scale cross-sectional and meta-analytic studies show that leisure-time physical activity—especially activities such as walking, running, cycling, and team sports—are more strongly associated with reduced odds of depression and improved mental health than aggregate measures like total metabolic equivalents (METs) alone [73,74,75].

Importantly, sex-specific differences have been identified: vigorous-intensity activity is more strongly associated with reduced depression and anxiety in men, while walking and moderate-intensity activity confer greater emotional well-being benefits in women [76]. These findings challenge the traditional approach of using aggregate weekly METs as the sole metric and support the use of activity-specific and domain-specific measures in both research and clinical recommendations [74,76].

Furthermore, the dose–response relationship is curvilinear, with the greatest mental health benefits observed when moving from inactivity to low or moderate levels of activity, and diminishing returns at higher doses [9,77]. The qualitative aspects of activity—such as social interaction, outdoor environment, and personal preference—also mediate these effects [73,74,78].

In summary, future research and clinical practice should prioritize nuanced, activity-specific, and sex-sensitive physical activity metrics rather than relying solely on aggregate METs, to optimize mental health outcomes.

The lack of a significant association between body mass index (BMI) and depressive or anxiety symptoms in medical student cohorts is consistent with emerging evidence that the well-established U-shaped relationship between BMI and mental health in the general population may not generalize to more homogenous, young adult populations such as medical students. Large population-based studies consistently demonstrate a U-shaped or dose-dependent association between BMI and depression, with both underweight and obesity conferring increased risk for depressive symptoms, and sometimes anxiety, across diverse adult and adolescent samples [63,64,65,66,79,80,81]. However, these associations are often attenuated or absent in younger, healthier, and more socioeconomically homogenous cohorts.

Recent studies specifically in medical students suggest that while overweight and obesity may be associated with increased depressive and anxiety symptoms, these relationships are frequently mediated by behavioral and psychosocial factors such as academic burnout and internet addiction, rather than BMI alone [22]. In young adult twin cohorts, elevated BMI is associated with poorer physical well-being and, to a lesser extent, depressive symptoms, but not with anxiety or social well-being, further supporting the notion that BMI-mental health associations are context-dependent and may be less pronounced in healthy, educated young adult [67,82].

These findings support the importance of prioritizing behavioral and psychosocial risk factors—such as academic stress, burnout, and maladaptive coping behaviors—over BMI alone when assessing mental health risk in medical students and similar cohorts. This approach aligns with the evolving understanding that mental health in young adults is multifactorial and less directly tied to BMI than in the general population.

The medical literature consistently demonstrates a strong and independent association between poor sleep quality and increased depressive and anxiety symptoms, particularly in high-stress populations such as those exposed to chronic stressors or pandemic conditions [54,62,83,84,85,86]. Sleep quality is repeatedly shown to be a central determinant of mental health, with bidirectional relationships observed: poor sleep predicts future depression and anxiety, and these symptoms also worsen sleep quality over time [54,62].

Importantly, several studies indicate that sleep quality functions as a distinct and independent pathway to mental health, rather than merely mediating the effects of physical activity on depression and anxiety. For example, while physical activity is associated with improved sleep and reduced depressive symptoms, mediation analyses reveal that sleep only partially mediates this relationship, and in some cases, the mediation effect disappears after adjusting for baseline depressive symptoms [68,69]. Other studies show that the negative impact of poor sleep quality on depressive symptoms is amplified in physically inactive individuals, but sleep and physical activity exert independent effects [6]. Furthermore, emotion regulation and adaptive coping strategies are independently associated with mental health outcomes, and their benefits are contingent on sleep quality, further supporting the notion that sleep is a unique pathway [84,87].

In summary, sleep quality is a central and independent determinant of mental well-being in high-stress populations, and its effects on depression and anxiety are not fully explained by its relationship with physical activity. This underscores the importance of directly targeting sleep quality in interventions aimed at improving mental health.

The most significant practical implication of our findings is the need for sex-specific recommendations for physical activity in mental health interventions for medical students. The study showed that for female students, walking time was a significant protective factor against depressive symptoms, while for male students, higher overall physical activity (Total METs) was more strongly associated with fewer depressive symptoms. This suggests that “one-size-fits-all” advice on physical activity may be less effective. By tailoring interventions, such as promoting accessible, light-intensity activities like walking for female students and encouraging vigorous exercise for male students, medical programs could achieve higher engagement and better mental health outcomes. This approach moves beyond generic guidance and offers a more personalized, evidence-based strategy to address the unique needs of different student groups.

A significant practical implication of this study is the clear roadmap it provides for universities and medical schools to develop evidence-based interventions that focus on modifiable lifestyle factors. Our findings, which found no significant association between BMI and depressive or anxiety symptoms, align with current evidence that suggests poor sleep quality and insufficient physical activity are highly prevalent and strongly associated with adverse health outcomes in university students, independent of BMI alone [88,89,90,91]. Therefore, prevention programs should prioritize improving sleep quality and increasing physical activity levels, as these are actionable and have demonstrated benefits for both physical and mental health in university settings. This approach is consistent with recommendations from leading professional societies, such as the American Heart Association, which highlights the effectiveness of behavioral interventions focused on physical activity and sleep health for promoting well-being and risk reduction [92]. Interventions targeting sleep hygiene and regular physical activity are recommended as first-line strategies, as students with better sleep quality and higher levels of moderate-to-vigorous physical activity (MVPA) show improved physical fitness and lower stress perception, regardless of BMI status.

Another important practical implication is that walking can be promoted as a simple, low-cost intervention. The finding that walking is a protective factor against depressive symptoms is consistent with a recent meta-analysis showing that even modest increases in daily step counts are associated with a reduction in depressive symptoms [12]. This reinforces the idea that “something is better than nothing”. Medical schools can leverage this by encouraging students to integrate short walks into their routines, perhaps through “walk-and-talk” study groups or by promoting the use of stairs over elevators. These interventions are supported by evidence that structured walking programs can reduce depression and improve well-being in student populations [93,94]. This makes beneficial behavior accessible to all students, regardless of their fitness level or access to exercise facilities and aligns with World Health Organization recommendations for regular activity to improve health and reduce the risk of depression [95].

Romanian medical education involves dense curricula and frequent high-stakes assessments, which may heighten psychological distress and shape coping behaviours. In this setting, a visible gym-oriented trend among some male students (bodybuilding/strength training) is time-intensive and may be difficult to reconcile with academic demands, whereas female students more often adopt lower-barrier activities such as walking. Campus features, a dispersed, multi-site teaching layout with constrained parking capacity—encourage active commuting between buildings, consistent with the comparatively high baseline walking observed. These contextual features help interpret the sex-specific patterns in our data while avoiding overgeneralization.

### 4.3. Strengths and Limitations

This investigation possesses several significant methodological and conceptual strengths. A key strength is its contribution to the underrepresented body of literature on the mental health of Romanian medical students, offering a unique regional perspective that expands upon findings from Western cohorts. The use of validated and widely accepted psychometric instruments, such as the International Physical Activity Questionnaire (IPAQ) and the Hospital Anxiety and Depression Scale (HADS), ensures the reliability and comparability of our measurements across studies. Furthermore, our comprehensive modeling strategy, which integrated a broad spectrum of demographic, behavioral, and psychological variables, allowed for a more detailed understanding of their complex interrelationships. The formal sex-stratified analysis is a particularly robust feature of our design, as it uncovered distinct sex-specific associations between physical activity types and mental health outcomes, thereby challenging the utility of a uniform, aggregate approach to physical activity promotion.

Despite these strengths, the study is subject to several important limitations. The cross-sectional design fundamentally precludes any causal inferences; while we observed significant associations, the directionality of these relationships—for example, whether a lack of physical activity contributes to depression or vice versa—cannot be determined. The data, being self-reported, introduces the potential for recall and social desirability biases, which could compromise the accuracy of measurements for physical activity, sleep quality, and screen time. Additionally, our analytic sample size, while adequate for primary analyses, may have been underpowered to detect subtle or curvilinear effects, such as the BMI-mental health relationship, particularly within a population that exhibits a narrow BMI range. Finally, the study’s focus on a specific regional cohort limits the generalizability of our findings to medical students in other cultural or academic contexts, highlighting the need for replication in diverse populations.

## 5. Conclusions

This cross-sectional study of Romanian medical students underscores the significant impact of specific lifestyle behaviors on mental health, with distinct patterns emerging for depressive and anxiety symptoms. Our findings confirm that physical activity, particularly walking, is a protective factor against depressive symptoms. This relationship is not uniform, as the psychological benefits appear to be sex-specific: female students showed a significant link between walking and reduced depressive symptoms, while male students benefited more from overall higher-intensity physical activity.

The study also reinforces the critical importance of sleep quality, which emerged as a strong and independent predictor for both depressive and anxiety symptoms. In contrast, we found no significant association between body mass index (BMI) and mental health outcomes when controlling other factors, suggesting that focusing on modifiable behaviors like sleep and physical activity may be more relevant for this specific population. The lack of a significant mediating effect through sleep or social media use indicates that these factors are not the primary mechanisms linking walking to mental health in this cohort. The protective effects of physical activity are thus likely direct or mediated by other unmodelled physiological or psychosocial factors, such as self-efficacy or neurotrophic processes.

In summary, these findings provide a clear, evidence-based roadmap for developing targeted, sex-specific interventions within medical education. By promoting both improved sleep hygiene and tailored physical activity—emphasizing walking for female students and higher-intensity activities for male students—universities can implement effective and accessible strategies to mitigate the high burden of depressive and anxiety symptoms in their medical student population.

## Figures and Tables

**Table 1:** Descriptive statistics of the analytic sample (*n* = 277).

Variable	N	Mean
Age (years)	277	23.61
BMI (kg/m^2^) [excluding ≤12 or >40]	274	22.41
Total MET-min/week	277	3309.43
PSQI	277	7.2
HADS-D	277	4.4
HADS-A	277	6.42

**Table 2:** Sleep and Physical Activity Descriptives (Overall).

Metric	Value
PSQI (mean ± SD)	7.20 ± 3.43
PSQI (median [IQR])	7 [5–9]
Poor sleep (PSQI > 5), *n*/N (%)	186/277 (67.15%)
Total MET-min/week (mean ± SD)	3309 ± 4260
Total MET-min/week (median [IQR])	2079 [132–4551]
Meets WHO minimum (≥600 MET-min/week), *n*/N (%)	190/277 (68.59%)
Walking time, min/day (mean ± SD)	80.20 ± 68.3
Walking time, min/day (median [IQR])	80 [0–135]

**Table 3:** IPAQ category distribution (Overall).

IPAQ Category	*n*	%
Low	101	36.50
Moderate	79	28.50
High	97	35.00

**Table 4:** Sleep and physical activity by sex with between-sex *p*-values.

Metric	Male	Female	Between-Sex *p*
PSQI (mean ± SD)	7.57 ± 2.83	7.06 ± 3.62	0.114
Total MET (mean ± SD)	4152 ± 6082	3002 ± 3328	0.987
Walking min/day (mean ± SD)	70.4 ± 74.1	83.7 ± 65.9	0.1151
Poor sleep prevalence (PSQI > 5)	78.40%	63.10%	0.0239
Meets WHO minimum (≥600 MET-min/week)	60.80%	71.43%	0.124

Notes: Poor sleep defined as PSQI > 5. WHO minimum = ≥150 min moderate or ≥75 min vigorous weekly or equivalent ≈ ≥600 MET-min/week [34]. IPAQ categories per standard thresholds. Between-sex *p* from Mann–Whitney U (continuous) and chi-square (prevalence).

**Table 5:** Spearman correlations between BMI, physical activity, and mental health outcomes.

Pair	ρ	*p*-Value
BMI—HADS-D	0.09	0.15
BMI—HADS-A	−0.07	0.26
Total MET—HADS-D	−0.19	0.0015
Total MET—HADS-A	0.00	0.98

**Table 6:** Mean HADS scores by IPAQ category (Low, Moderate, High).

IPAQ Category	HADS-D Mean	HADS-A Mean
Low (*n* = 101)	5.19	6.46
Moderate (*n* = 79)	3.92	6.25
High (*n* = 97)	3.96	6.51

**Table 7:** Ordinary least squares (OLS) regression for depressive symptoms (HADS-D).

Predictor	β	SE	t	*p*	95% CI
Intercept	7.8522	2.152	3.649	<0.001	[3.615, 12.089]
BMI	−0.0104	0.099	−0.105	0.917	[−0.205, 0.185]
BMI^2^	0.0004	0.001	0.298	0.766	[−0.002, 0.003]
Total MET	−0.0001	0.000	−1.805	0.072	[−0.000,0.000]
PSQI TOTAL	0.3675	0.065	5.673	<0.001	[0.240, 0.495]
RSES	−0.0491	0.031	−1.560	0.120	[−0.111, 0.013]
Social Media Time	−0.3770	0.152	−2.476	0.014	[−0.677, −0.077]
Gaming Time	−0.1604	0.107	−1.493	0.137	[−0.372, 0.051]
Age	−0.0451	0.028	−1.605	0.110	[−0.100, 0.010]
Female	−1.6214	0.503	−3.227	0.001	[−2.611, −0.632]

Model fit: *n* = 277, R^2^ = 0.236, Model F-test *p* < 0.001. Predictors included: BMI, BMI^2^, Total MET-min/week, PSQI, RSES, social-media time, gaming time, age, and sex (Female). Coefficients are unstandardized β (SE), with 95% CI and two-sided *p*-values.

**Table 8:** Ordinary least squares (OLS) regression for anxiety symptoms (HADS-A).

Predictor	β	SE	t	*p*	95% CI
Intercept	6.9559	2.584	2.692	0.008	[1.868, 12.044]
BMI	−0.2253	0.119	−1.895	0.059	[−0.459, 0.009]
BMI^2^	0.0028	0.001	1.949	0.052	[−0.001, 0.006]
Total MET	0.0001	0.000	0.913	0.362	[−0.000, 0.000]
PSQI TOTAL	0.3971	0.078	5.104	<0.001	[0.244, 0.550]
RSES	−0.1259	0.038	−3.327	0.001	[−0.200, −0.051]
Social Media Time	0.3328	0.183	1.820	0.070	[−0.027, 0.693]
Gaming Time	−0.0498	0.129	−0.386	0.700	[−0.304, 0.204]
Age	0.0194	0.034	0.576	0.565	[−0.047, 0.086]
Female	0.6273	0.604	1.039	0.300	[−0.561, 1.815]

Model fit: *n* = 277, R^2^ = 0.212, Model F-test *p* < 0.001. Predictors included: BMI, BMI^2^, Total MET-min/week, PSQI, RSES, social-media time, gaming time, age, and sex (Female). Coefficients are unstandardized β (SE), with 95% CI and two-sided *p*-values.

**Table 9:** Spearman correlations between walking indicators and HADS outcomes.

	Depressive Symptoms (HADS-D)	Anxiety Symptoms (HADS-A)
Days walked per week	ρ = −0.19 *p* = 0.002 *	ρ = −0.11 *p* = 0.067
Minutes walked per day	ρ = −0.15 *p* = 0.012 *	ρ = −0.03 *p* = 0.634
Total walking METs	ρ = −0.15 *p* = 0.010 *	ρ = −0.04 *p* = 0.526

* *p* < 0.05.

**Table 10:** Correlations Between Physical Activity, BMI, and Symptoms by Sex.

Metric	Male	Female
BMI—Score HADS-D	ρ = −0.02, *p* = 0.8539	ρ = 0.08, *p* = 0.2281
BMI—Score HADS-A	ρ = 0.02, *p* = 0.8672	ρ = −0.06, *p* = 0.3684
Total MET—Score HADS-D	ρ = −0.35, *p* = 0.0021 *	ρ = −0.12, *p* = 0.0759
Total MET—Score HADS-A	ρ = −0.03, *p* = 0.7874	ρ = 0.01, *p* = 0.8675
Minutes Walked—Score HADS-D	ρ = −0.14, *p* = 0.2239	ρ = −0.14, *p* = 0.0443 *
Minutes Walked—Score HADS-A	ρ = −0.05, *p* = 0.6796	ρ = −0.02, *p* = 0.7294
Total Walk MET—Score HADS-D	ρ = −0.15, *p* = 0.1932	ρ = −0.13, *p* = 0.0595
Total Walk MET—Score HADS-A	ρ = −0.03, *p* = 0.7707	ρ = −0.04, *p* = 0.5346

* *p* < 0.05.

## Data Availability

The dataset generated during the current study is available in the Zenodo repository at https://doi.org/10.5281/zenodo.17073937 (accessed on 6 October 2025).

## Abbreviations

The following abbreviations are used in this manuscript:

BMI	body mass index
CI	confidence interval
GAD-7	Generalized Anxiety Disorder 7-item scale
HADS	Hospital Anxiety and Depression Scale
HADS-A	Hospital Anxiety and Depression Scale—Anxiety subscale
HADS-D	Hospital Anxiety and Depression Scale—Depression subscale
HC3	Heteroscedasticity-Consistent Covariance Matrix Estimator 3
IPAQ	International Physical Activity Questionnaire
IPAQ-SF	International Physical Activity Questionnaire—Short Form
IQR	interquartile range
MET	metabolic equivalent of task
MVPA	moderate-to-vigorous physical activity
OLS	ordinary least squares
PA	physical activity
PHQ	Patient Health Questionnaire
PSQI	Pittsburgh Sleep Quality Index
RSES	Rosenberg Self-Esteem Scale
SD	Standard Deviation
SE	Standard Error
WHO	World Health Organization
